# Fluid Leak From the Spinal-Epidural Puncture Site: Is That Cerebrospinal Fluid?

**DOI:** 10.7759/cureus.47211

**Published:** 2023-10-17

**Authors:** Rui Torres, Rita P Sa, Ana S Cruz, Juliana L Cruz, Lúcia V Maria

**Affiliations:** 1 Anesthesiology, Hospital de Braga, Braga, PRT

**Keywords:** spinal-epidural anesthesia, anesthesia and pain, neuraxial anesthesia complications, c-section, cutaneous cerebrospinal fluid fistula

## Abstract

We present the case of a 30-year-old parturient who underwent a combined spinal-epidural for an elective cesarean section and subsequently experienced fluid leakage at the puncture site. The fluid analysis indicated a glucose level of 57 mg/dL, which initially raised suspicion of a cerebrospinal fluid fistula. However, an MRI revealed no significant abnormalities, and the patient remained asymptomatic. We aim to highlight that various fluids can emerge from a neuraxial puncture site, including cerebrospinal fluid, interstitial fluid due to edema, or residual local anesthetic. While glucose measurement has been used for diagnosing cerebrospinal fluid leakage, its reliability is questionable. More dependable diagnostic tests can be used such as the measurement of beta-trace protein or beta-2-transferrin or MRI.

## Introduction

Neuraxial techniques are widely regarded as the preferred choice for obstetric anesthesia [1]. Among these techniques, the combined spinal-epidural (CSE) approach is frequently used to promptly initiate anesthesia and allow for the sustained management of anesthesia and postoperative analgesia via epidural [1]. With an incidence of 0.16%, cutaneous cerebrospinal fluid (CSF) fistula is a rare complication that manifests as a fluid leak through the insertion site, which can be accompanied by symptoms of CSF hypotension and may also lead to complications such as meningitis among others [2]. However, not all insertion site fluid leaks should be interpreted as a CSF fistula [3], and appropriate tests should be conducted for diagnosis.

## Case presentation

A 30-year-old parturient with no significant medical history was scheduled for an elective cesarean section due to a history of previous cesarean sections. The patient underwent a pre-anesthetic consultation in which a regional anesthesia technique was proposed and accepted by the patient.

With the patient in a seated position, the L3-L4 space was identified using the loss of resistance technique with a 16-gauge Touhy needle. Once the epidural space was identified (at 3.5 cm), a 27-gauge pencil point needle was introduced through the previous needle, and a subarachnoid block was performed using 7 μg of hyperbaric bupivacaine and 2 μg of sufentanil. The 27-gauge needle was removed, and an epidural catheter was introduced, with the tip positioned 7 cm from the skin.

The epidural catheter was used intraoperatively, administering 30 mg of ropivacaine at a concentration of 7.5 mg/mL due to prolonged surgical time caused by an inadvertent bladder laceration, which was promptly repaired. The patient remained hospitalized for 48 hours, receiving 16 mg of ropivacaine at 2.0 mg/mL with 10 μg of sufentanil via epidural bolus every four hours for postoperative pain management, without any sensory or motor deficits, headaches, or other complications.

On the third postoperative day, after the removal of the epidural catheter, clear fluid was observed at the site of the CSE puncture (Video 1).

**Video 1:** Liquid aspiration with a 2 mL syringe.

The patient did not experience headaches, nausea, sensory deficits, or any significant complaints. Capillary blood glucose was measured (137 mg/dL), and the analysis of the fluid showed a glucose level of 57 mg/dL, resulting in a blood/fluid ratio of 0.4. Considering the suspicion of a fistula, a sterile compressive dressing was applied, and an MRI was requested, which the patient underwent the following day.

On the fourth postoperative day, the patient no longer exhibited fluid leakage at the puncture site, and the dressing remained clean and dry. The MRI did not reveal any significant abnormalities. The patient remained asymptomatic and was discharged on the same day. The patient was re-evaluated in a postoperative appointment one week later and did not present any complaints or clinical signs.

## Discussion

Diagnosing the underlying cause of fluid drainage from a spinal-epidural puncture site can be challenging. Several cases have been reported of asymptomatic patients diagnosed with cutaneous CSF fistulas [4,5]. The absence of symptoms typically associated with CSF hypotension, such as headaches, nausea, and vomiting, adds complexity to the management of these patients. It is imperative to recognize that not every fluid emerging from a neuraxial technique puncture site comprises CSF but can be interstitial fluid due to edema, a seroma, or even residual local anesthetic [3].

Many researchers, due to its accessibility, have pondered diagnosing CSF fistulas through glucose testing of the draining fluid [4-6]. However, this approach is limited by its low sensitivity, and it may not be the most accurate method for diagnosing CSF leakage [7,8]. An alternative and more reliable diagnostic strategy entails evaluating the presence of beta-trace protein or beta-2-transferrin, proteins specifically found in CSF [7,8]. These tests have been employed in similar cases to diagnose CSF fistulas [4].

In this case report, we present a patient with fluid drainage from the puncture site following a spinal-epidural technique. The fluid exhibited a glucose level of 57 mg/dL, corresponding to a blood/fluid ratio of 0.4. However, neuroimaging results ruled out the presence of a CSF fistula. Unfortunately, it was not possible to perform tests for the presence of beta-trace protein or beta-2-transferrin. Nonetheless, the imaging examination conducted demonstrates sufficient accuracy for diagnosing cerebrospinal fluid fistulas [9,10].

## Conclusions

The utility of glucose measurement or glucose strip tests as diagnostic modalities for CSF cutaneous fistulas is fraught with limitations, being unreliable for definitive diagnosis. Instead, a more robust diagnostic approach entails the utilization of MRI or the quantification of beta-trace protein or beta-2-transferrin.
